# A Novel Combination of γ-Tocopherol-Rich Mixture of Tocopherols and Ascorbic Acid Restores Fertility in Cases of Tyrosine Nitration-Associated Male Infertility in Mice

**DOI:** 10.3390/antiox9070613

**Published:** 2020-07-13

**Authors:** Eleonora Scarlata, Maria C. Fernandez, Cristian O’Flaherty

**Affiliations:** 1 Department of Surgery (Urology Division), McGill University, Montréal, QC H4A 3J1, Canada; eleonora.scarlata@mail.mcgill.ca (E.S.); maria.c.fernandez@mail.mcgill.ca (M.C.F.); 2 The Research Institute, McGill University Health Centre, Montréal, QC H4A 3J1, Canada; 3 Department of Pharmacology and Therapeutics, McGill University, Montréal, QC H3G 1Y6, Canada

**Keywords:** reactive oxygen species, vitamin E, vitamin C, spermatozoa, fertilization, lipid peroxidation

## Abstract

Infertility is an important health problem that affects up to 16% of couples worldwide. Male infertility is responsible for 50% of the cases. Currently, a physical examination, hormone profiling and the evaluation of two consecutive semen samples (to determine the sperm concentration, motility, morphology and, in very few cases, sperm DNA integrity) are the sole tools that physicians have to evaluate infertility in men. Antioxidant therapy is often used to improve sperm quality and function in infertile men. However, there are controversial results regarding the efficacy of these treatments. *Prdx6^−/−^* male mice are subfertile, displaying significant oxidative damage in the lipids, proteins and DNA of their spermatozoa. Here, we used *Prdx6^−/−^* male mice to test whether a novel combination of tocopherols that contained 60% γ-tocopherol and ascorbic acid could restore their fertility. These mice were fed with the supplemented (Vit. Mix) or control diets. To assess sperm quality, we determined the motility, levels of lipid peroxidation, DNA oxidation and tyrosine nitration in the spermatozoa. The number of pups sired by the *Prdx6^−/−^* mice fed with the Vit. Mix diet was higher than that sired by the males fed with the control diet, and the pups’ mortality was lower. The sperm quality was improved in the males fed with the supplemented diet. We concluded that treatment with a supplement composed of tocopherols and rich in γ-tocopherol and ascorbic acid is effective in restoring fertility in cases where oxidative stress and high levels of tyrosine nitration are associated with male infertility.

## 1. Introduction

Male infertility is of great medical issue in humans because approximately 16% of couples worldwide are affected by infertility, and in 50% of these cases, the causes can be ascribed to the man [1]. Environmental pollutants, chemicals, drugs, smoke, toxins, radiation and diseases that have adverse effects on fertility have in common oxidative stress [2,3,4,5,6]. The presence of high levels of reactive oxygen species (ROS) in semen has been reported in 25–87% of infertile patients [7,8,9].

Male idiopathic infertility has a prevalence of 23% [10]. We found that patients with idiopathic infertility had reduced amounts of peroxiredoxin 6 (PRDX6) in spermatozoa compared to healthy donors [11]. Of note, thiol-oxidized PRDX6 (inactive) levels and the presence of high molecular mass complexes (containing the inactive PRDX6-SO_2_) [12] are present in the spermatozoa in 90% of idiopathic infertile men [11]. Due to the reduced amounts of the PRDX6 protein and its oxidation status, very little antioxidant protection (less than 20%) remains in the spermatozoa. This reduced antioxidant protection could be a possible cause of the impairment of sperm function and poor DNA quality observed in these patients [11].

Our studies demonstrated that human spermatozoa displayed higher levels of DNA oxidation when treated with specific inhibitors of PRDX6 calcium-independent phospholipase A_2_ (iPLA_2_) activity (1-hexadecyl-3-(trifluoroethyl)-sn-glycero-2-phosphomethanol lithium (MJ33)) than the non-treated controls or sperm incubated with inhibitors of PRDX1–5 (conoidin A), thioredoxin reductase (TRD; auranofin) or GSTπ (ezatiostat), or in which GSH was depleted with ethacrynic acid [13]. By establishing dose–response curves, we determined which concentrations of these inhibitors were toxic by assessing sperm viability by flow cytometry using calcein-AM and found that human spermatozoa are more sensitive to the inhibition of PRDX6 iPLA_2_ (with MJ33) than that of 2-Cys PRDXs (with conoidin A) [13]. Moreover, the toxic lipid peroxidation product 4-hydroxynonenal (4-HNE) was significantly increased by the treatment with MJ33 but not with conoidin A, suggesting that it is the PRDX6 iPLA_2_ activity that protects spermatozoa against lipid peroxidation [13]. This protection solely by PRDX6 is important because 4-HNE is mutagenic and responsible for damage to DNA [14]. We also found that PRDX6 peroxidase activity alone protects the spermatozoon against increased levels of peroxynitrite (ONOO^–^) [13]. Because ONOO^−^ induces lipid peroxidation [15], PRDX6, through its two enzymatic activities, is the protector of the paternal genome against lipid peroxidation. Altogether, these findings strongly suggest an essential role of PRDX6 in the protection of human spermatozoa against oxidative stress and offer a possible cause for the impaired sperm function in idiopathic infertile patients.

*Prdx6^−/−^* male mice are subfertile; they produce 50% fewer viable pups than wild-type males and show sperm of low quality with low motility and significant oxidative damage [16]. We previously demonstrated that fertilization rates were reduced by 80% when using *Prdx6^−/−^* compared to wild type (WT) spermatozoa, and *Prdx6^−/−^* spermatozoa were unable to produce blastocysts (pre-implantation embryos) in vitro [17]. Because of the poor reproductive performance of *Prdx6^−/−^* males under in vivo and in vitro conditions, this model is ideal for studying how ROS affect male fertility and the search for a successful treatment.

Antioxidant therapy is a common clinical strategy for improving sperm quality and function in infertile men. There are controversial results regarding the efficacy of these treatments [9]. In the particular case of treatment with vitamins E and C, there are controversial data supporting the beneficial effect on the achievement of live births [9,18]. Tocopherols (the active form of vitamin E) scavenge ROS, and ascorbic acid converts the tocopheryl radicals (oxidized tocopherol generated by ROS) [19] to tocopherol for the re-cycling of vitamin E. As mentioned above, PRDX6 peroxidase activity is the unique scavenger of ONOO^–^ in human spermatozoa [13]. Although both α- and γ-tocopherol can scavenge H_2_O_2_ and organic peroxides, γ-tocopherol is a more efficient scavenger of ONOO^–^ and of lipophilic electrophiles capable of inducing lipid peroxidation than α-tocopherol [15,20,21], the most commonly used vitamin E form in antioxidant supplement preparations.

Here, we used *Prdx6^−/−^* male mice to determine whether dietary supplementation with a combination of tocopherols rich in γ-tocopherol and ascorbic acid restored their fertility.

## 2. Materials and Methods

### 2.1. Animals and Dietary Treatment

Wild type (WT, C57Bl6/J) and *Prdx6^−/−^* male mice were produced from our colonies at the Research Institute, McGill University Health Centre (Montreal, QC, Canada). The C57Bl6/J breeder pairs were purchased from Charles River (Laval, QC, Canada). The *Prdx6^−/−^* mouse model was generated by Dr. Y. Ho in collaboration with Dr. A. Fisher at the University of Pennsylvania (Philadelphia, PA, USA) [22,23]. The mice were maintained on a 14L/10D cycle. Food and water were provided ad libitum. All procedures were performed according to the regulations of the Canadian Council for Animal Care and were approved by the Animal Care Committees of McGill University and the McGill University Health Centre (animal protocol #2009-5656).

Wild type and *Prdx6^−/−^* male mice were fed with a base diet (control) or a base diet supplemented with 0.3% mixed tocopherols (containing 60% γ-tocopherol) [24] and a 1.75 g of ascorbic acid/kg diet. Both diets were produced by ResearchDiets (New Brunswick, NJ, USA). We fed male mice (6 weeks old) (*n* = 8 per group) ad libitum either with the control diet or the treatment diet (Vit. Mix diet) for 43 days (the period to produce a full cycle of spermatogenesis and epididymal maturation in the mouse). Then, we mated these males with age-matched WT females (ratio, 1:1) and determined the reproductive outcomes (the litter size, unsuccessful mating, the weight of the pups at birth and the pup mortality rate) of three consecutive matings and the sperm quality (motility, lipid peroxidation, DNA oxidation and tyrosine nitration) in males euthanized after the last mating. The males continuously received the Vit,. Mix diet to assure high levels of vitamins until the end of the matings, the WT females were caged overnight, and the presence of a vaginal plug was considered indicative of successful mating. The number of matings, litter size and weight of the pups at weaning were recorded. At the end of the experiment, the males were euthanized to harvest the testes and spermatozoa for experiments.

### 2.2. Tissue Collection and Preparation

Male mice fed with the control or Vit. Mix diets were euthanized and weighed, and the testes, epididymes and spermatozoa were collected. Immediately after collection, both cauda epididymes were placed in 300 μL of Biggers, Whitten and Wittingham (BWW) medium composed of 91.5 mM NaCl, 4.6 mM KCl, 1.7 mM CaCl_2_, 1.2 mM KH_2_PO_4_, 1.2 mM MgSO_4_, 25 mM NaHCO_3_, 5.6 mM D-glucose, 0.27 mM sodium pyruvate, 44 mM sodium lactate and 20 mM HEPES for 10 min at 37 °C to let the sperm swim out. Then, the spermatozoa in the BWW medium were collected, aliquots were used to determine the sperm motility, and the rest of the samples were stored at −80 °C until further use.

### 2.3. Determination of Sperm Motility and Epididymal Maturation

We used a computer-assisted sperm analysis system (CASA) with the Sperm Vision HR software version 1.01 (Minitube, Ingersoll, ON, Canada) to evaluate the total and progressive sperm motility. A total of 200 spermatozoa were examined for each sample to determine the percentages of sperm total and progressive motility. We determined the percentage of cytoplasmic droplet retention to evaluate epididymal maturation, as performed previously [25].

### 2.4. Determination of Lipid Peroxidation

We determined the levels of lipid peroxidation in spermatozoa by flow cytometry using a BODIPY 581/591 C11 probe (Thermo Fisher Scientific, Toronto, ON, Canada) as previously described [17]. Sperm samples from *Prdx6^−/−^* and WT mice were incubated with 5 µM BODIPY 581/591 C11 in HEPES balanced saline (HBS, pH 7.4) for 30 min at 37 °C in the dark. A sperm aliquot with 40 µM ferrous sulphate (FeSO_4_) in HBS, incubated for 2 h at 37 °C, was used as the positive control. Ten thousand or more spermatozoa were analyzed for each sample using a MACSQuant Analyzer flow cytometer (Miltenyi Biotec, Inc., Auburn, CA, USA) equipped with an argon laser (488 nm) and 585/625 nm filter. The levels of lipid peroxidation are expressed as the relative intensity of the green fluorescence/red + green fluorescence.

### 2.5. Determination of DNA Oxidation

The levels of 8-hydroxy-2’-deoxyguanosine (8-OHdG) were determined by immunocytochemistry, as we previously reported [16,25]. Frozen spermatozoa were centrifuged to remove the PBS, thawed at 37 °C, and resuspended in PBS supplemented with 40 μM DTT, 1 μM EDTA and 1% SDS and incubated for 5 min at room temperature. Then, smears were prepared and allowed to air dry. Dried cells were permeabilized with 100% methanol at −20°C, rehydrated with PBS supplemented with 0.1% Triton X-100 (PBS-T), and blocked with 5% horse serum in PBS-T for 30 min at room temperature. After washing with PBS-T, slides were incubated overnight at 4 °C with an anti-8-OHdG antibody (catalog # SMC-155, StressMarq Biosciences Inc, Victoria, BC, Canada) at a 1:100 dilution. Then, the cells were washed and incubated with goat anti-mouse antibody conjugated with Alexa Fluor Pluss 555 (Thermo Fisher Scientific, Toronto, ON, Canada) at a 1:2000 dilution. Positive controls were made by incubating spermatozoa with 5 mM H_2_O_2_ and 1 MM FeSO_4_.7H_2_O in BWW medium [16,25]. The specificity of the primary antibody was confirmed by incubating smears of the sperm samples with the primary antibody previously incubated with 8-OHdG (Abcam Inc, Toronto, ON, Canada) at a concentration 1000 times higher than that of the primary antibody for 1 h at 20 °C, as performed previously [26]. Fluorescent signals were analyzed under an epifluorescence microscope (Zeiss Axiophot, Germany). All the images were captured with a digital camera (Retiga1300, QImaging, Burnaby, BC, Canada) and digitized with the Northern Eclipse digital imaging software, version 6.0 (Empix Imaging, Mississauga, ON, Canada). The results are expressed as the percentages of spermatozoa with a positive signal. At least 200 spermatozoa were counted per sample.

### 2.6. Determination of Tyrosine Nitration in Spermatozoa

We determined the levels of tyrosine nitration in spermatozoa from WT and *Prdx6^−/−^* males fed with the control and Vit. Mix diets by immunocytochemistry, as previously performed, with modifications [27]. After thawing, spermatozoa were smeared onto slides and allowed to dry. Then, the cells were permeabilized with 100% methanol at −20 °C, rehydrated with PBS-T and blocked with 5% goat serum for 30 min at room temperature. The sperm smears were washed again with PBS-T and incubated overnight at 4 °C with the anti-nitro-tyrosine antibody (catalog # ab61392, Abcam Inc, Toronto, ON, Canada) at a 1:100 dilution. Fluorescent signals were analyzed under an epifluorescence microscope (Zeiss Axiophot, Germany). All the images were captured with a digital camera (Retiga1300, QImaging, Burnaby, BC, Canada) with the same time exposure and digitized with the Northern Eclipse digital imaging software, version 6.0 (Empix Imaging, Mississauga, ON, Canada). The results are expressed as the percentages of spermatozoa with positive signals and as the relative fluorescence intensity (RFI). To determine the RFI, digitized grayscale pictures were analyzed with the Image J software V1.42q (National Institutes of Health, Bethesda, MD, USA). The mean gray value of the background was subtracted from the values measured in the spermatozoa. The results are expressed as relative units of RFI. At least 200 spermatozoa were counted per sample.

### 2.7. Statistical Analysis

**Figure 1:**
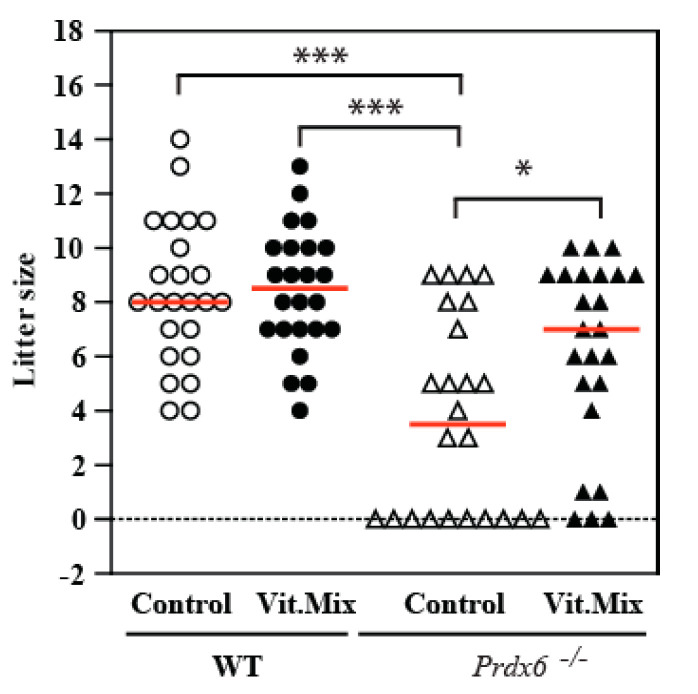
Number of pups sired by wild type (WT) or *Prdx6^−/−^* males fed with control or Vit. Mix diets. Each circle or triangle corresponds to one litter from WT or *Prdx6^−/−^* males, respectively. Open symbols correspond to males fed with the control diet, and black symbols correspond to males fed with the Vit. Mix diet. A horizontal red line represents the median of each group. * *p* ≤ 0.05; *** *p* ≤ 0.001, two-way ANOVA and Tukey tests.

**Figure 2:**
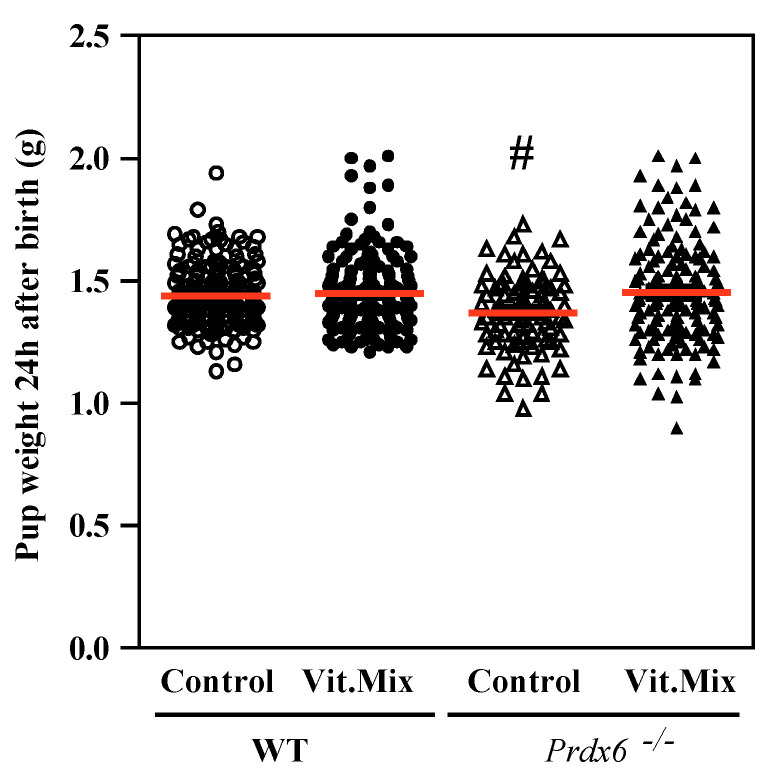
Pup weight at 24 h after birth. Each circle or triangle corresponds to one litter from WT or *Prdx6^−/−^* males, respectively. Open symbols correspond to males fed with the control diet, and black symbols correspond to males fed with the Vit. Mix diet. The mjedian of each group is represented by a horizontal red line. # means smaller than all the other groups (*p* ≤ 0.05, two-way ANOVA and Tukey tests).

All the results are presented as mean ± SEM, except for those in Figure 1 and Figure 2, where the medians are included in the dot plot graphs. The normality of the data and homogeneity of variances were determined with the Shapiro–Wilk and Bartlett tests, respectively. We used the two-way ANOVA and Bonferroni tests (to assess changes due to genotype and diet) and chi-square tests as appropriate using GraphPad Prism 5 (GraphPad Software, Inc., San Diego, CA, USA) to determine statistical differences among groups.

## 3. Results

### 3.1. Prdx6^−/−^ Males Supplemented with the Vit. Mix Diet Have Similar Reproductive Outcomes to Wild Type Mice

*Prdx6^−/−^* male mice fed with the Vit. Mix diet showed similar body, testis and epididymis weights but produced a lower percentage of non-fertile matings than the *Prdx6^−/−^* males fed with the control diet (Table 1). The *Prdx6^−/−^* male mice fed with the Vit. Mix diet produced higher numbers of pups than the *Prdx6^−/−^* males fed with the control diet, similar number to that produced by the WT mice (fed with either of the two diets) (Figure 1). Moreover, there were no significant differences in the percentages of non-fertile matings observed in the *Prdx6^−/−^* mice fed with the Vit. Mix diet and the WT mice (fed with the control or the supplemented diet). When we consider only the fertile matings, the litter size was affected by the genotype but not by the diet.

**Figure 3:**
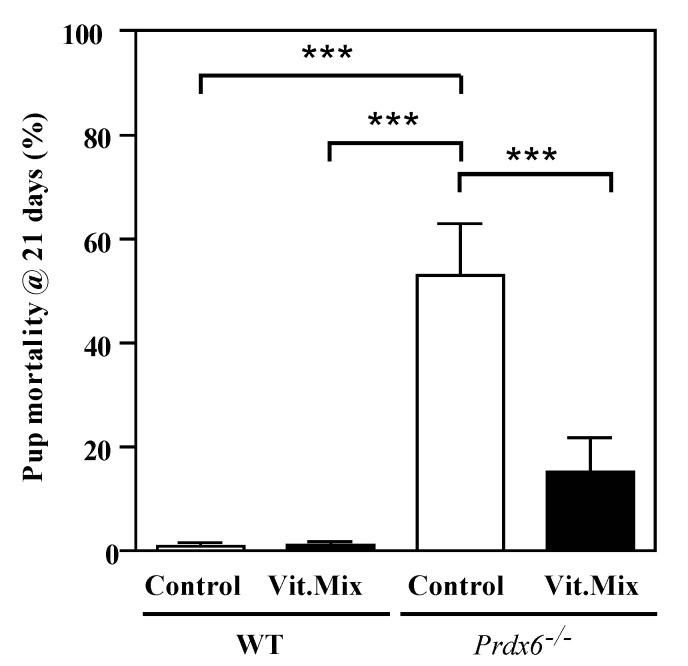
Mortality of pups sired by WT or *Prdx6^−/−^* males fed with control (open bars) and Vit. Mix (black bars) diets, respectively. *** *p* ≤ 0.001, two-way ANOVA and Tukey tests.

We observed that the pups sired by the *Prdx6^−/−^* males fed with the control diet were significantly smaller than those sired by the males of the other three groups (Figure 2). The mortality rates at weaning time (21 days) were the lowest in the pups sired by the *Prdx6^−/−^* males fed with the Vit. Mix diet. Interestingly, the supplementation with the Vit. Mix diet prevented the death of pups, and pups sired by the *Prdx6^−/−^* males fed the supplemented diet showed no significant differences in mortality rates from WT controls (Figure 3).

### 3.2. Vit. Mix Diet Improved Sperm Quality in Prdx6^−/−^ Male Mice

**Figure 4:**
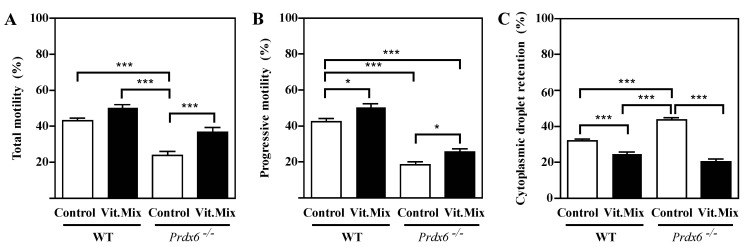
Motility and epididymal maturation of spermatozoa from WT or *Prdx6^−/−^* males fed with the control (open bars) and Vit. Mix (black bars) diets, respectively. Total (**A**) and progressive (**B**) motility and (**C**) cytoplasmic droplet retention, a marker of immature spermatozoa. * *p* ≤ 0.05; *** *p* ≤ 0.001, two-way ANOVA and Tukey tests.

Spermatozoa recovered from *Prdx6^−/−^* mice fed with the Vit. Mix diet showed higher total motility than those from those fed with control diet, with values similar to those obtained for the WT mice fed with either diet (Figure 4a). Progressive motility in the *Prdx6^−/−^* mice was lower than that in the WT controls, but the Vit. Mix diet partially and significantly restored the progressive motility of the *Prdx6^−/−^* mice compared to the control diet (Figure 4b). The percentage of cytoplasmic droplet retention, an indicator of immature spermatozoa and thus impaired epididymal maturation, was significantly lower in those mice fed with the Vit. Mix diet than in the controls, with no significant differences from the WT controls (Figure 4c).

### 3.3. Oxidative Stress in Spermatozoa Was Reduced by the Vit. Mix-Supplemented Diet

**Figure 5:**
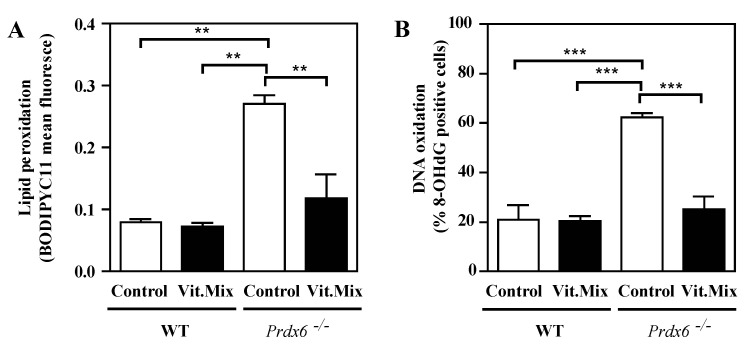
Lipid peroxidation (**A**) and DNA oxidation (**B**) in spermatozoa from WT or *Prdx6^−/−^* males fed with control (open bars) and Vit. Mix (black bars) diets, respectively. (**A**) Lipid peroxidation measured by flow cytometry using a BODIPYC11 fluorescent probe. (**B**) DNA oxidation determined by the levels of 8-hydroxy-2’-deoxyguanosine (8-OHdG) in spermatozoa according to immunocytochemistry. ** *p* ≤ 0.01; *** *p* ≤ 0.001, two-way ANOVA and Tukey tests.

We determined the level of oxidative stress according to lipid peroxidation, DNA oxidation and tyrosine nitration levels in spermatozoa from all groups. We found that the levels of lipid peroxidation determined with the BODIPYC11 probe (Figure 5a) and DNA oxidation determined by the levels of 8-OGHdG (Figure 5b) were significantly lower in the spermatozoa from the *Prdx6^−/−^* mice fed with the vitamin-supplemented diet than in the *Prdx6^−/−^* mice fed with the control diet. Moreover, there were no significant differences in the levels of lipid peroxidation and DNA oxidation when comparing the *Prdx6^−/−^* males fed with the Vit. Mix diet and WT controls.

**Figure 6:**
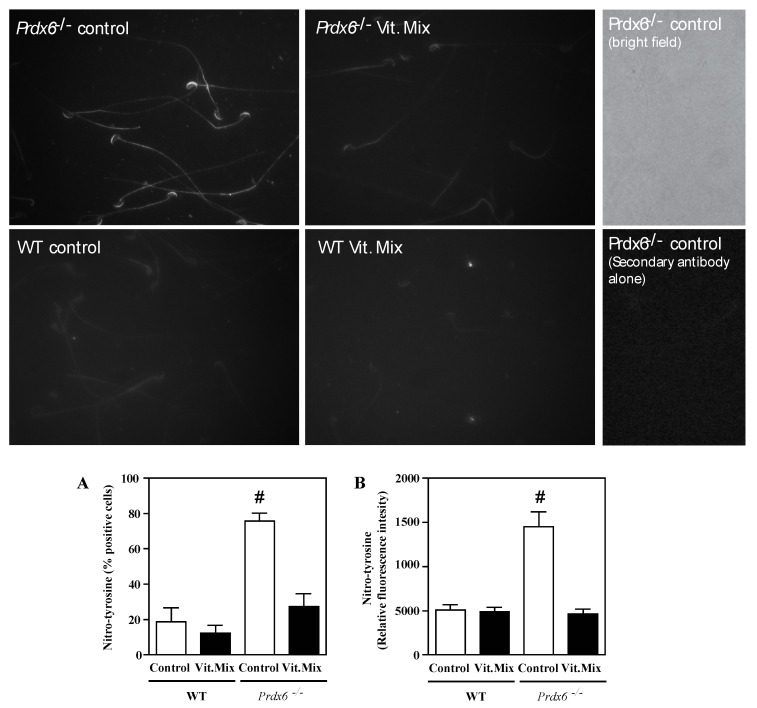
Tyrosine nitration in spermatozoa from WT or *Prdx6^−/−^* males fed with control (open bars) and Vit. Mix (black bars) diets, respectively. Unspecific binding was ruled out by incubating *Prdx6^−/−^* spermatozoa with secondary antibody alone (right panels). Percentages (**A**) and relative fluorescence intensity (**B**) were determined by immunocytochemistry, and analysis of the fluorescence was performed using the ImageJ software. # means higher than all the other groups. *p* ≤ 0.001, two-way ANOVA and Tukey tests.

Strong tyrosine nitration labelling was observed in the acrosome regions, midpieces and principal pieces of spermatozoa from *Prdx6^−/−^* males fed with the control diet (Figure 6). The *Prdx6^−/−^* males fed with the Vit. Mix and the WT males had a similar pattern of tyrosine nitration distribution in their spermatozoa but to a significantly lesser extent. Indeed, the percentage of spermatozoa with positive labelling and the fluorescence intensity observed were significantly higher in the spermatozoa from the *Prdx6^−/−^* males fed with the control diet than in the other groups (Figure 6C,D).

Despite the primary effects of the genotype already observed and of the treatment, we observed interactions between the genotype and the type of diet in all the parameters studied except for the sperm motility and number of pups, indicating that infertile males with significant damage due to oxidative stress and tyrosine nitration in their sperm benefit from the antioxidant treatment (Table 2). In the case of the number of pups, the *p* value of 0.06 indicates the existence of a trend of interaction.

## 4. Discussion

Oxidative stress has been identified as a culprit in 25–87% of male infertility cases [9]. However, the benefits of antioxidant therapy for treating male infertility are still controversial. For more than a decade, a series of systematic reviews have concluded that there are insufficient quality data to indicate the benefit of using antioxidant therapy in infertile men to improve live birth rates and pregnancy rates [9,28,29]. One of the causes of this low-quality evidence for beneficial effects is the inappropriate selection of male participants. In most, if not all, randomized-controlled studies, including those in these reviews, the selection of patients did not include the assessment of whether oxidative stress was present in the semen. Thus, in some cases of male infertility, antioxidant therapy will not be beneficial because the causes of infertility are not associated with oxidative stress. Although the evaluation of oxidative stress markers seems obvious based on the high percentage of infertile men that have oxidative stress in the semen [7,8,9], the practicality of including assays for the evaluation of ROS, antioxidants and oxidative stress markers is challenging, since these assays are difficult to implement widely at fertility clinics.

*Prdx6^−/−^* males are subfertile, and their spermatozoa are highly sensitive to oxidative stress [16]. Moreover, *Prdx6^−/−^* spermatozoa failed to produce viable embryos after in vitro fertilization [17]. The high levels of lipid peroxidation, and oxidation of proteins and DNA, along with low motility and an incapacity to fertilize oocytes in vitro, are abnormal outcomes that are frequently found in infertile men. Previously, we reported that PRDX6 is present in low amounts in spermatozoa from idiopathic infertile men [11]. Furthermore, PRDX6 was highly thiol-oxidized and, therefore, inactive in the sperm of these patients.

In this study, we used the *Prdx6^−/−^* mouse model to determine whether supplementation with a novel combination of a γ-tocopherol-rich mixture of tocopherols and ascorbic acid could restore fertility. The combination of vitamins used in this study successfully restored fertility in *Prdx6^−/−^* males, since these males sired a higher number of pups with higher weights at birth and a lower mortality rate than those pups sired by *Prdx6^−/−^* males fed with the control diet (Figure 1, Figure 2 and Figure 3). These data indicate that supplementation using an antioxidant formulation enriched with γ-tocopherol is safe, producing healthy offspring. At a cellular level, the supplementation with the vitamin mix improves the sperm quality of *Prdx6^−/−^* males. Indeed, a higher percentage of motility and lower levels of DNA oxidation and tyrosine nitration were observed in spermatozoa from those *Prdx6^−/−^* males fed with the supplemented diet than in those from those fed with the control diet.

The paternal genome’s integrity is essential to ensure healthy embryo development. Even when spermatozoa contain significant DNA damage, they can fertilize oocytes, but embryo development is severely affected [30]. We observed that in infertile men with varicocele or idiopathic infertility with high levels of lipid peroxidation in the seminal plasma and spermatozoa, the amount of DNA damage depends on the level of thiol-oxidized PRDX6 in their spermatozoa [11]. The thiol oxidation in PRDX6 promotes enzymatic inactivation and, therefore, the incapacity of PRDX6 to scavenge ROS. The lack of PRDX6 generates high levels of DNA fragmentation and oxidation in mouse spermatozoa [16]. The inhibition of PRDX6 peroxidase’s and iPLA_2_ activities promotes a dose-dependent decrease in sperm viability associated with an increase in DNA oxidation [13]. Thus, the absence or inactivation of PRDX6 is a plausible cause of sperm DNA damage in infertile men.

The peroxidation of the lipids in the sperm plasma membrane is associated with low motility and infertility. PRDX6 iPLA_2_ activity is involved in the repair of peroxidized membranes [31]. *Prdx6^−/−^* spermatozoa display higher levels of lipid peroxidation than WT controls, and WT spermatozoa incubated with MJ33 have similarly high levels as those from the knockout mice, indicating that PRDX6 iPLA_2_ activity is necessary to protect the sperm plasma membrane against oxidative stress and allow normal sperm motility [17,25]. Human spermatozoa also require PRDX6 iPLA_2_ to maintain low levels of lipid peroxidation and assure sperm motility viability and fertilization ability [13,32].

Along with the iPLA_2_ activity, PRDX6 displayed peroxidase activity that is important for the antioxidant defenses in the cell. The inhibition of PRDX6 peroxidase activity is associated with an increase in the endogenous production of ONOO^−^ upon treating healthy human spermatozoa with a NO^•^ donor (DA-NONOate) and an inhibitor of complex III of the mitochondrial electron transport chain (antimycin A) [13]. Moreover, the DA-NONOate treatment was sufficient to increase the levels of tyrosine nitration in human spermatozoa [27]. High levels of tyrosine nitration were observed in spermatozoa from asthenozoospermic infertile men [33,34]. These previous findings and our present results (Figure 6) suggest that tyrosine nitration is associated with impairment of sperm motility and that PRDX6 peroxidase activity is the primary scavenger of ONOO^−^ and, by maintaining low levels of this ROS, assures normal sperm motility [13,27,33,34].

Noteworthily, peroxynitrite can induce the formation of lipid hydroperoxides in an iron-free fashion [15]. The inhibition of PRDX6’s peroxidase activity produces an increase in endogenous ONOO^−^ and 4-HNE in human spermatozoa [13]. Moreover, the mitochondrial superoxide production and loss of mitochondrial membrane potential depend on the level of 4-HNE [13,35]. These findings indicate that ONOO^—^ participates in the production of lipid peroxidation in human spermatozoa.

Although the γ-tocopherol form is a powerful antioxidant, it has been unexplored as an antioxidant treatment for male infertility. Low levels of γ-tocopherol but not of α-tocopherol have been reported in semen from infertile men with varicocele [36]. Low levels of these tocopherols were found in the blood and seminal plasma of a population of infertile patients comprising men with asthenozoospermia, asthenoteratozoospermia and oligoasthenozoospermia [37]. These studies are the first to demonstrate the associations of low levels of γ-tocopherol with low motility or varicocele in infertile patients and support our suggestion of treating male infertility with a combination of tocopherols enriched with γ-tocopherol and ascorbic acid. Indeed, the supplementation of the diet fed to the *Prdx6^−/−^* males with the Vit. Mix resulted in better reproductive outcomes than those seen in the *Prdx6^−/−^* males fed with the control diet. This improvement was reflected not only by the increased number of healthy pups produced (Figure 1, Figure 2 and Figure 3) but also by better sperm quality, as indicated by the high sperm motility (Figure 4) and low levels of lipid peroxidation, DNA oxidation and tyrosine nitration (Figure 5 and Figure 6) observed in the *Prdx6^−/−^* male mice that received the vitamin supplement.

Tocopherols (the active form of vitamin E) scavenge ROS and are converted into tocopheryl radicals. Then, ascorbic acid converts the tocopheryl radicals to tocopherols for the re-cycling of vitamin E [19]. As mentioned above, PRDX6 peroxidase activity is the unique scavenger of ONOO^–^ in human spermatozoa [13]. Although both α- and γ-tocopherol can scavenge H_2_O_2_ and organic peroxides, γ-tocopherol is a more efficient scavenger of ONOO^–^ and of lipophilic electrophiles that generate lipid peroxidation than α-tocopherol [15,20,21], the most commonly used vitamin E form in antioxidant supplement preparations. This efficiency in removing ONOO^–^ is essential for protecting the spermatozoon against lipid peroxidation and preventing the formation of 4-hydroxynonenal (4-HNE), a toxic by-product of the peroxidation of lipids. This is important because 4-HNE can form protein and DNA adducts, inactivating enzymes and promoting mutations in the paternal genome, respectively [14,38].

As mentioned above, lipid peroxidation impairs sperm motility [39,40], and its product, 4-HNE, inactivates the enzyme succinate dehydrogenase in sperm [35]. The inactivation of this enzyme promotes increased mitochondrial levels of superoxide anion, which leads to a reduction in motility and the promotion of apoptosis-like changes in human spermatozoa. Since ONOO^−^ promotes lipid peroxidation [15], treatment with an antioxidant therapy rich in γ-tocopherol may restore fertility in those men with significant levels of tyrosine nitration in their semen that are unable to father a child. Further studies should be done to determine whether an antioxidant supplement rich in γ-tocopherol will be beneficial for treating infertility in men.

## 5. Conclusions

In conclusion, our study using the *Prdx6^−/−^* model is a proof of concept that a supplement containing a γ-tocopherol-enriched tocopherol mixture and ascorbic acid supplement are useful in restoring fertility in this laboratory model. Indeed, infertile men with a deficiency in PRDX6 peroxidase activity (or the PRDX6 protein) in their spermatozoa are at higher risk from the damage caused by ONOO^−^-induced lipid peroxidation [15], even if they take α-tocopherol supplements. It is important to highlight that without an appropriate strategy to identify idiopathic infertile men with oxidative stress in their semen, any antioxidant therapy implemented will probably fail, a situation that is reflected in the comprehensive reviews published in the last decade [9,28,29]. With such diagnostic tools that can easily be incorporated into the clinical evaluation of the infertile patient, it is possible to design randomized controlled trials with a large number of subjects, where the inclusion criteria include evidence of ongoing oxidative stress. These studies will confirm whether antioxidant therapy is useful for the treatment of cases of idiopathic infertility when high levels of ROS are the cause of the disease.

## Figures and Tables

**Table 1:** Body, testis and epididymis weights and fertile matings.

Genotype	Diet	Body Weight (g)	Testis Weight (% of BW)	Epididymis Weight (% of BW)	Non-Fertile Matings (%)
WT	Control	27.9 ± 0.1	0.68 ± 0.01	0.27 ± 0.01	0% (0/24)
	Vit. Mix	28.1 ± 0.2	0.7 ± 0.021	0.28 ± 0.02	0% (0/24)
*Prdx6^−/−^*	Control	27.7 ± 0.4	0.73 ± 0.02	0.28 ± 0.01	41.6% (10/24) *
	Vit. Mix	25.3 ± 0.3	0.67 ± 0.03	0.26 ± 0.01	12.5% (3/24)

* Means significantly higher than all others (Chi-Square, *p* ≤ 0.05).

**Table 2:** Complete summary (*p*-values) of statistical analysis by two-way ANOVA.

	Number of Pups	Pup Weight	Pup Mortality	Total Motility	Progressive Motility	Cytoplasmic Droplet	Lipid Peroxidation	DNA Oxidation	NitroY (% cells)	NitroY (Relative Intensity)
Genotype	<0.0001	0.018	<0.0001	<0.0001	<0.0001	0.0036	<0.0001	0.0002	0.0002	<0.0001
Diet	0.04	0.0009	0.0022	<0.0001	0.0002	<0.0001	<0.0001	0.0009	0.0009	<0.0001
Genotype x diet	0.06	0.027	0.002	0.19	0.86	<0.0001	<0.0001	0.0023	0.006	<0.0001
